# Lithium metal deposition/dissolution under uniaxial pressure with high-rigidity layered polyethylene separator

**DOI:** 10.1039/d0ra02788j

**Published:** 2020-05-07

**Authors:** Shogo Kanamori, Mitsuhiro Matsumoto, Sou Taminato, Daisuke Mori, Yasuo Takeda, Hoe Jin Hah, Takashi Takeuchi, Nobuyuki Imanishi

**Affiliations:** Department of Chemistry for Materials, Graduate School of Engineering, Mie University Tsu Mie 514-8507 Japan taminato@chem.mie-u.ac.jp +81-59-231-9478 +81-59-231-9968; LG Chem, LG Chem R&D Campus Daejeon 188, Munji-ro, Yuseong-gu Daejeon 34122 Korea

## Abstract

The effects of mechanical uniaxial pressure and deflection of the separator on the electrochemical deposition of lithium metal were investigated. Instead of dendritic lithium growth without pressure, a much more dense and compact deposition can be achieved when pressure is applied to the cells during the lithium deposition process. This morphology is due to the formation of granular lithium followed by the generation of new lithium nuclei on the cathode surface. The improved lithium plating/stripping behavior in the cells under mechanical pressure yielded a 10% higher coulombic efficiency than cells without pressure. However, the cycle life is shortened with pressures higher than 1.39 MPa; therefore, there is an upper limit for improvement of the electrochemical characteristics near 1.39 MPa. The morphology of electrodeposited lithium becomes flatter with a large amount of electrodeposition under pressure when the number of polyethylene separators is increased to five due to the increase in the stiffness of the layered separators. Furthermore, high coulombic efficiency cycling by pressurization was increased to twice that for one separator sheet. Application of the optimal strength pressure and use of more inflexible separators are thus effective methods to control the microscopic morphology of electrodeposited lithium and improve the cycle performance of the lithium metal anode.

## Abstract 2

The use of a high rigidity separator and application of an appropriate amount of pressure are effective approaches to control lithium metal growth and improve its cycle performance.

## Graphical abstract

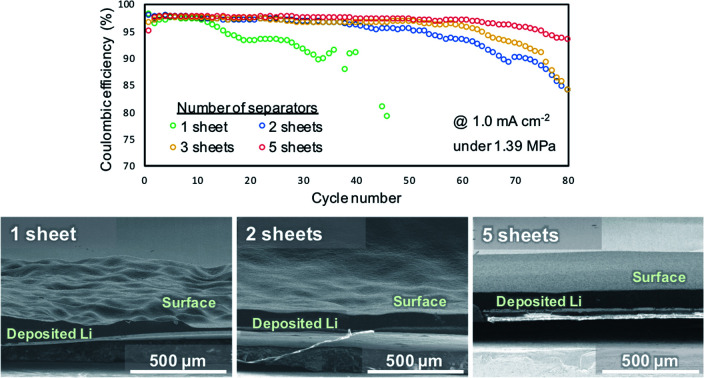

## 1 Introduction

Lithium ion batteries have been widely used as the power source for most advanced electronic devices such as laptops and mobile phones that are indispensable in our daily lives. These batteries have also recently been used for electric vehicles and power storage system applications to address environmental problems.^1–5^ Therefore, the establishment of high energy density systems with long-term stability is required. The lithium ion battery is composed of a positive electrode (cathode), negative electrode (anode), organic electrolyte, and separator. Although the energy densities are improving year by year, the current system that uses a Li(Ni,Mn,Co)O_2_ cathode and graphite anode has almost reached the theoretical limit.^6–8^ Lithium metal is considered as a candidate material for the anode of high energy secondary batteries because of its low electrode potential (−3.04 V *vs.* standard hydrogen electrode (SHE)) and high specific capacity (3860 mA h g^−1^).^9–11^ However, the safety problems and poor cycle life hinders its use in practical applications.^12–15^ When lithium metal is used as the anode, a lithium metal precipitation reaction occurs during the charge process. At this time, the current is partially concentrated on the electrode due to the inhomogeneous shape of the deposition surface and a thick solid electrolyte interphase (SEI), which results in the formation lithium dendrites with high surface area. This causes penetration to the cathode side and excessive decomposition of the organic electrolyte, which leads to the internal short-circuiting of the cell and depletion of the organic electrolyte.^16–20^ Therefore, it is most important to suppress dendrite formation when a lithium metal anode is used in a practical rechargeable system.

Various chemical approaches have been studied to solve this drawback, including the used of mixed-electrolyte salts and -solvents and the addition of organic and inorganic additives.^21–23^ According to the report by Ding *et al.*, the formation of dendritic crystals can be suppressed by the addition of cations with lower standard reduction potentials than that of lithium ions, such as cesium and rubidium ions.^24^ Togasaki *et al.* reported that the cycle performance of lithium plating/stripping reaction was stabilized by the use of LiNO_3_ as the electrolyte salt.^25^ Such studies have revealed that the electrolyte components and additives can lead to the suppression of dendritic growth and improvement of the cycle performance of the lithium metal anode. However, it is difficult to provide practical cycle performance of lithium metal anodes because the additives and electrolyte components continue to be consumed during the lithium metal electrodeposition/dissolution reactions.^26^

On the other hand, a physical approach that takes advantage of the relatively soft nature of lithium metal has been noticed in recent years.^27–29^ One approach is the application of mechanical pressure against the lithium metal anode, which influences the morphology and electrochemical performance of the cell. Wilkinson *et al.* reported that mechanical pressure has a profound effect on lithium plating morphology and cyclability, by which short-circuiting can be avoided.^28^ Yin *et al.* reported that application of pressure to the cell induced the deformation of deposited lithium, which improved the coulombic efficiency and cycle life.^29^ However, the electrodeposition mechanism under pressure and the pressure dependence of the electrochemical and morphological characteristics of lithium remain unclear. Therefore, to gain more understanding of the importance of pressure on lithium metal batteries, it is necessary to investigate the relationship between the application of pressure and the morphological changes and cycle performance of lithium metal anodes. The mechanical properties of the separators (polymer membrane and inorganic solid electrolyte) are also important parameters for dendrite suppression and long-cycle life. A hard separator is effective in suppressing lithium dendrite growth. It was reported that the use of a hard separator or a separator modified with ceramic and organic materials can inhibit the growth of lithium perpendicular to the substrate, whereby cycle performance is improved.^30–33^ In addition, there has been focus on not only on the strength but also the rigidity of separators. Monroe and Newman reported the relationship between the physical properties of solid polymer electrolytes and the stability of the lithium/electrolyte interface based on linear elasticity theory.^34^ Accordingly, for a polymer material with a Poisson's ratio similar to poly(ethylene oxide), interfacial roughening is mechanically suppressed when the separator shear modulus is approximately twice that of lithium metal. Furthermore, considering the practical level of the current density and capacity of the lithium metal anode, microscopic change of the shape, such as the flatness of the lithium metal anode, is also an important issue with respect to homogeneous electrode reaction of the lithium anode. In particular, the contribution of the mechanical strength of a separator should be pronounced in a cell under pressure due to strong contact between the electrodeposited lithium and the separator.

In this study, we focused on the influence of external pressure to a cell and the rigidity (mechanical strength rather than the hardness) of a layered separator on the electrodeposition mechanism of lithium metal. It is necessary to analyze whether the physical morphology or manner of lithium growth are changed under pressure to clarify the electrodeposition mechanism in more detail. Therefore, we especially analyzed the initial lithium growth process under pressure. In addition, the electrodeposition morphology and electrochemical behavior under wider pressurization conditions were investigated to determine the pressure threshold.

A cell with an electrolyte solution was used instead of a solid electrolyte in this experiment for ease of analysis of the morphology of electrodeposited lithium. Therefore, the number of polyethylene separator layers was changed to achieve greater rigidity. The effects of the external pressure and the rigidity of the separator layer on the lithium metal electrodeposition were evaluated with respect to the morphology and electrochemical characteristics.

## 2 Experimental

### 2.1. Assembly of pouch cells

**Fig. 1:**
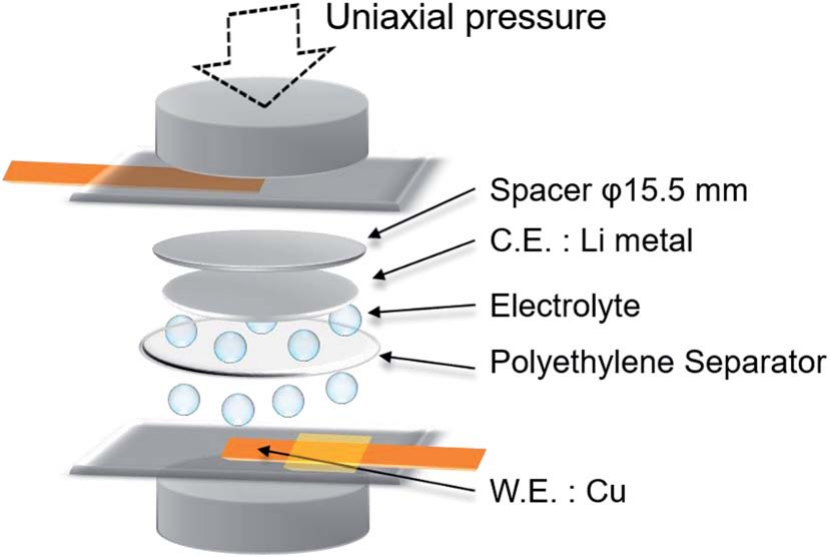
Structure of Li/Cu laminated cell with closed structure used in this study.

Electrochemical lithium plating/stripping was tested under uniaxial pressure using a laminated type cell with closed structure (Fig. 1). Lithium metal (electrode area: 0.785 cm^2^) was used as the anode, and copper metal (electrode area: 0.49 cm^2^) was used as the cathode. 50 μL of 1 mol dm^−3^ LiPF_6_ in a mixture of ethylene carbonate (EC) and diethyl carbonate (DEC) (1 : 1 v/v%) was used as the electrolyte. One sheet of 20 μm thick polyethylene separator (area: 2.01 cm^2^) was used as the separator when the influence of pressure was tested, whereas 1 to 5 sheets were used for the cell to investigate the effect of separator rigidity (deflection).

Uniaxial pressure was applied using a spring, and calculated from the spring constant (21.81 N mm^−2^ or 49.39 N mm^−2^), spring displacement (6–16 mm) and pressure area (15.5 mm diameter, the same as the spacer). The physical characteristics for the separator such as shape and electrolyte content is changed by the stress from applied pressure because of the elasticity of polyethylene material. Therefore, considering the stress relaxation of the separator in the cells, lithium plating and charge/discharge measurements were conducted after pressurization for 1 h.

### 2.2. Characterization of electrodeposited lithium

Lithium metal was plated on the copper substrate under various pressures, after which the cell was disassembled in an Ar-filled glove box and the electrode surface rinsed with EC/DEC (1 : 1 v/v%). The morphology of lithium electrodeposited with and without pressure was analyzed using scanning electron microscopy (SEM; Hitachi S-4800). The electrode was cut with a razor to observe the cross sectional morphology.

### 2.3. Electrochemical measurements

The cycle performance of the lithium plating/stripping reaction was investigated by charge/discharge measurements under each pressure. The current densities were 0.2–2.0 mA cm^−2^ and the charge cutoff voltage was 0.5 V (*vs.* Li/Li^+^). Charge and discharge processes were repeated every hour. Electrochemical impedance spectroscopy (EIS) measurements were conducted using a Li/Cu cell after the Li plating reaction on a copper electrode at 1.0 mA h cm^−2^ with an impedance/gain phase analyzer (Solartron 1260) and a potentiostat/galvanostat (Solartron 1287). EIS measurements were performed in the frequency range between 0.1 and 1.0 MHz with an amplitude of 10 mV at 25 °C. The pressure applied to the cell was maintained during EIS measurements. The time dependence of the EIS spectra was recorded at each pressure.

### 2.4. Effect of separator rigidity (deflection)

The relationship between the number of polyethylene separators and the deflection was analyzed by a three-point deflection test with an automatic horizontal servo-controlled test stand (JISC JSV-H1000) and accompanying software (JISC SOP-EG1). One to five sheets of polyethylene separator were layered in this experiment. Polyethylene separators cut to a width of 5 mm were fixed at a distance of 20 mm between the fulcrums and a load was then applied to the center (pressure area: 5 × 0.5 mm). The morphology and cycle characteristics of the lithium metal anode were investigated with various numbers of separator sheets using the Li/Cu pouch cell shown in Fig. 1. The cross-sectional morphology of separators and lithium plated by reaction on the copper electrode at 15 mA h cm^−2^ were analyzed after samples were cut with a razor. The cycle performance of the cells with various numbers of separator sheets under a pressure of 1.39 MPa at 1.0 mA cm^−2^ was analyzed by charge/discharge measurements.

## 3 Results and discussion

### 3.1. Effect of uniaxial pressure during lithium metal electrodeposition

**Fig. 2:**
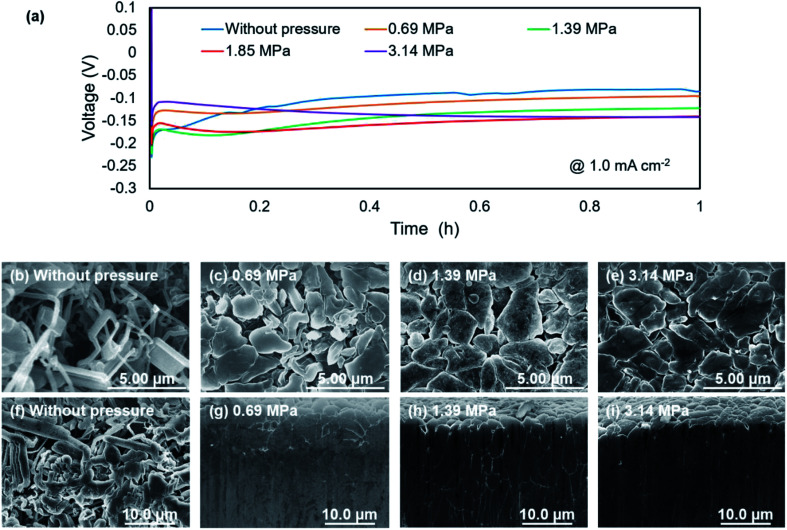
Characterization of lithium metal plating behavior on a copper substrate at 1.0 mA cm^−2^ under various uniaxial pressures. (a) First discharge curves for the plating reaction under various pressures. SEM images of electrodeposited lithium without pressure (b and f), and under pressures of 0.69 MPa (c and g), 1.39 MPa (d and h), and 3.14 MPa (e and i). Upper (b–e) and lower (f–i) panels are surface and cross-section images.

Fig. 2a shows discharge curves of the first lithium metal plating process on the copper substrate under various pressures. The dependence of the nucleation potential on the pressure could not be confirmed from the discharge behavior at the initial stage of lithium plating. When the capacity of exceeded 0.5 mA h cm^−2^, the polarization increased slightly with the applied pressure. Surface SEM images of electrodeposited lithium on the copper substrate (Fig. 2b–e) showed an elongated dendritic structure after operation without pressure, whereas densely packed particles were confirmed with the application of pressure. These results are similar to those previously reported for deposition morphology under pressure.^28,29^ The morphologies were observed over the entire surface of copper cathode for each pressure to confirm uniform pressure was applied over the entire electrode surface. No significant changes in morphology and density were observed between 1.39 MPa (Fig. 2d) and 3.14 MPa (Fig. 2e), which indicates that there is a certain threshold of pressure to the particle formation and densification of the electrodeposits. Fig. 2f–i show cross sectional SEM images of the electrodeposited lithium, which confirm how lithium is electrodeposited under pressure. Electrodeposition without the application of pressure resulted in lithium dendrites with high surface area (Fig. 2f), which causes side reactions and electrolyte decomposition. In contrast, densely packed lithium particles were observed for electrodeposition under pressure, although there were a few grain boundaries (Fig. 2g–i). These results are attributed to significant restriction of the growth space of lithium by uniaxial pressure during electrodeposition. Furthermore, the occurring creep phenomenon of lithium metal was observed around the 1 MPa stress by A. Masias *et al.*^35^ Thus, it is considered that the creep phenomenon occurred in this study, even under pressure at room temperature, because lithium is a very soft metal, which also led to lithium particles coming into contact with each other to form a dense morphology. This dense morphology could also suppress peeling of the electrodeposit, which is a common cause of capacity loss during lithium stripping.

**Fig. 3:**
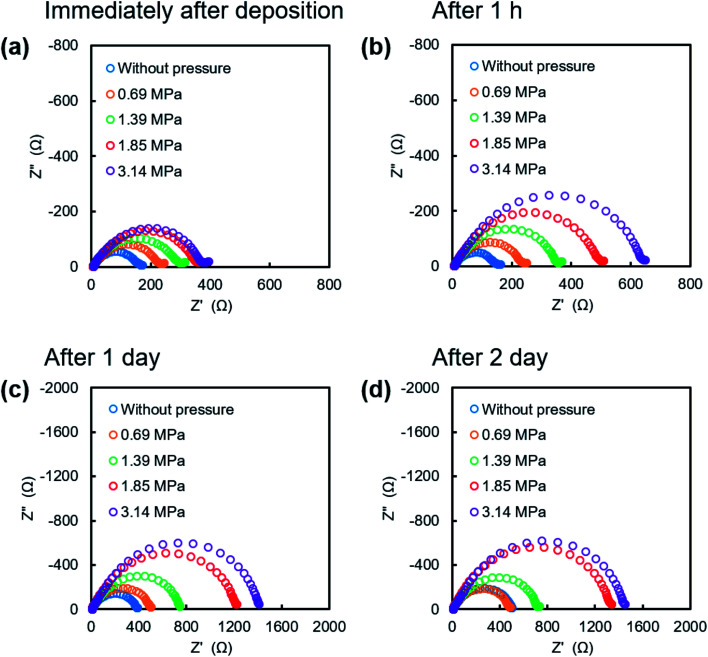
Nyquist plots of the Li/Cu pouch cell after the lithium plating reaction on the copper electrode at 1.0 mA h cm^−2^. EIS measurements were conducted (a) immediately, and after various storage times of (b) 1 h, (c) 1 day, and (d) 2 days.

**Fig. 4:**
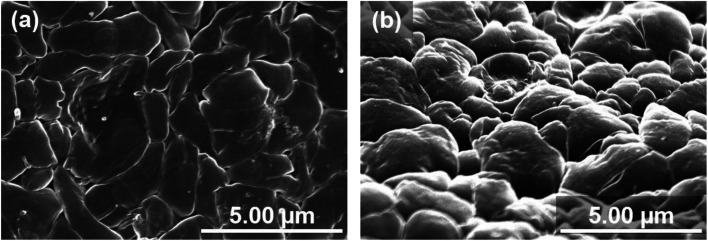
SEM image of the deposited lithium surface pressed with 3.14 MPa for 2 days. Lithium was electrodeposited on a copper substrate at a current density of 1.0 cm^−2^ for 1 hour. These were observed from (a) vertical and (b) oblique angles, respectively.

Nyquist plots obtained after electrodeposition under various applied pressures at 1.0 mA h cm^−2^ are shown in Fig. 3. EIS measurements were conducted after various storage times. The spectra contain a semicircle in the measurement range. The relaxation frequencies of all the semicircles show a 10^−6^ order, which are assigned to the resistance of the SEI membrane and charge transfer at lithium metal/electrolyte interface. A difference in interfacial resistance was observed with the applied pressure. Higher interfacial resistance was observed with an increase of the applied pressure. Compact and flat lithium metal with a low surface area was formed under pressure, as shown in Fig. 2, which results in the higher apparent interfacial resistance due to a small amount of reaction sites. However, the interface resistance was increased by applied pressure above 1.39 MPa, although no significant change in morphology could be confirmed. The time dependence of the resistance under pressure indicates that the interfacial resistance increased significantly after one hour when a pressure of 1.85 MPa was applied, whereas no significant change in resistance was observed below 1.39 MPa (Fig. 3b). Pressure applied at 3.14 MPa rapidly increased the interfacial resistance and pressure applied 1.85 MPa finally resulted in the same interfacial resistance as that at 3.14 MPa over time, although no change in the morphology of the lithium was observed with the storage time (Fig. 4). These results imply that the electrolyte solution is pushed to elsewhere in the cell and flatness of the separator is lost because the separator is compressed and elastically deformed under the pressure.^36^ Consequently, the contact between electrode and electrolyte solution is suppressed, which would lead the increase in interfacial resistance with storage time. Therefore, pressure below 1.39 MPa is effective to change the morphology of the electrodeposited lithium, which was also verified by charge/discharge tests.

### 3.2. Growth process of electrodeposited lithium under pressure

**Fig. 5:**
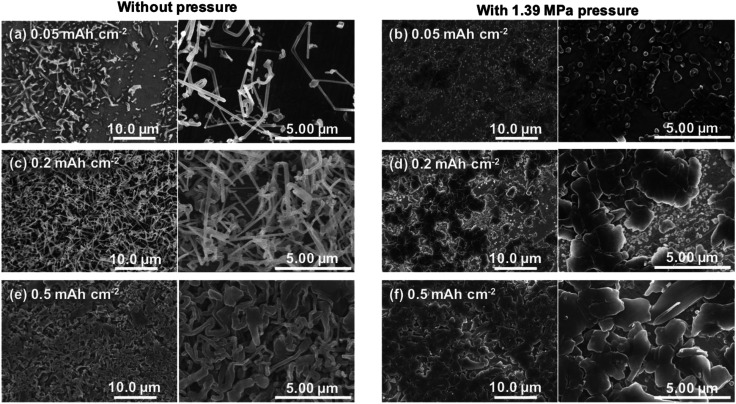
SEM images of copper electrode after lithium plating with and without uniaxial pressure. Electrodeposition at (a and b) 0.05 mA h cm^−2^, (c and d) 0.2 mA h cm^−2^ and (e and f) 0.5 mA h cm^−2^ was performed at a current density of 1.0 mA cm^−2^.

**Fig. 6:**
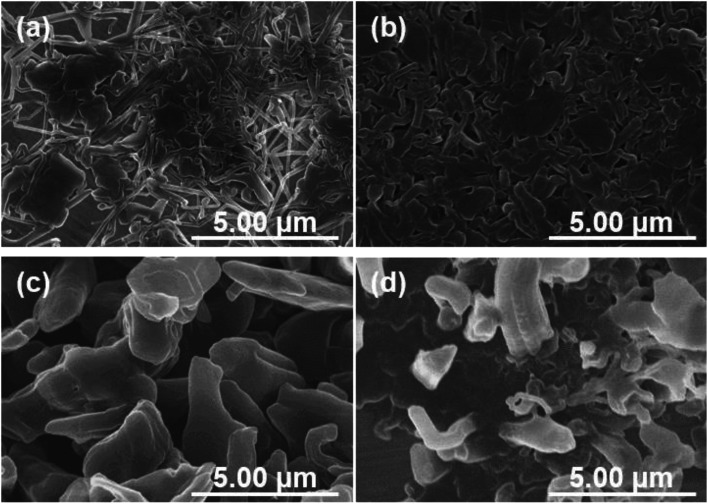
SEM images of the electrodeposited lithium when the pressure applied to the cell during lithium plating reaction. (a and b) Morphologies of electrodeposits plated at 1.0 mA cm^−2^ for 30 minutes under 1.39 MPa after lithium plating at 1.0 mA cm^−2^ for 30 minutes without pressure. (c and d) Those plated at 1.0 mA cm^−2^ for 30 minutes without pressure after lithium plating at 1.0 mA cm^−2^ for 30 minutes under 1.39 MPa.

The growth processes in lithium plating without pressure and with an applied pressure of 1.39 MPa were compared. Fig. 5 shows the morphologies of electrodeposits on the copper substrate after lithium plating with and without uniaxial pressure. The morphological changes depending on the amount of deposition were analyzed by adjusting the capacity during the plating reaction. In the initial stage of electrodeposition without pressure, elongated deposits were sparsely and unevenly distributed on the copper substrate (Fig. 5a). As the amount of electrodeposition increased, these grew into complex dendritic lithium with about 0.2 μm-thickness (Fig. 5c and e). In contrast, a large amount of small particles were observed at the initial stage of electrodeposition at 0.05 mA h cm^−2^ when the pressure was applied to the cell (Fig. 5b). For the range of capacity above 0.2 mA h cm^−2^, large plate-like particles with about 1–3 μm were observed with and some small ones between them (Fig. 5d and f). The lithium can plate at the space with less stress between large particles and grow to change its shape into larger domains in a horizontal direction by agglomeration with nearby particles. Electrochemical/mechanics model suggests that the load is carried at just the tallest asperities, where stresses reach tens of MPa, while most of the lithium surface feels no force at all. The lithium avoids plating at the tips of growing dendrites if there is sufficient local stress. Because the high stress makes separator pores to narrow, resulting in the extruded lithium ions plate elsewhere. And then creep ensures that grown lithium particles are gradually flattened.^37,38^ Our experimental results also suggest that the application of pressure limits a certain space between the electrode and separator for lithium growth, and lithium ions are plated with avoiding the tips of growing surface. This consideration is also supported by SEM images shown in Fig. 6. Lithium metal was electrodeposited at 1.0 mA cm^−2^ for 1 h using two pressure application methods. One method with no pressure applied during the first half of plating time, and then applied at 1.39 MPa during the second half (process 1). The other method was pressure applied at 1.39 MPa during the first half of the plating reaction time, and then released from the latter half of the plating time (process 2). Fig. 6a and b show SEM images of the electrodeposited lithium metal on the copper substrate by process 1 (without pressure → pressure applied at 1.39 MPa). No evidence of dendritic deposits was observed because of the pressure on the electrodeposited lithium during the reaction. The elongated lithium became thicker and more compact. The elongated shape of dendrites was broken and crushed due to the restriction of space by applied pressure during second half of plating time. Fig. 6c and d show SEM images of the electrodeposited lithium on the copper substrate by process 2 (pressure applied at 1.39 MPa → pressure released). When electrodeposition was performed after releasing the pressure from the middle of electrodeposition, dendritic growth was confirmed in local parts of the deposits. These results indicate that application of pressure is important to limit dendrite formation because the applied pressure continues to limit the lithium growth space.

### 3.3. Effect of pressure on charge/discharge behavior

**Fig. 7:**
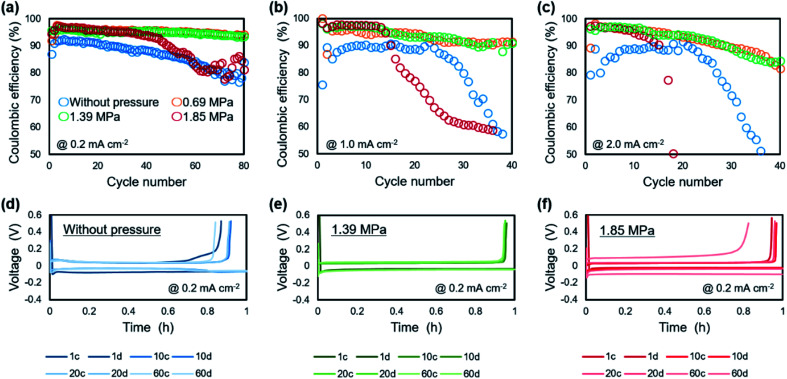
Electrochemical performance of lithium metal plating/stripping on a copper electrode under various uniaxial pressures. Coulombic efficiency at current densities of (a) 0.2 mA cm^−2^, (b) 1.0 mA cm^−2^ and (c) 2.0 mA cm^−2^. Charge/discharge curves of the Li/Cu cell at 0.2 mA cm^−2^ (d) without pressure, and under pressures of (e) 1.39 MPa and (f) 1.85 MPa.

The relationship between the strength of the applied pressure and cycle performance was investigated by charge/discharge measurement. Fig. 7 shows cycle dependence of coulombic efficiencies and plating/stripping curves with each cycle, when operated under various uniaxial pressures. At a current density of 0.2 mA cm^−2^ (Fig. 7a), the coulombic efficiency of the cell without pressure decreased monotonically with cycling from an initial 90%. The cells under pressures of 0.69 MPa and 1.39 MPa maintained a coulombic efficiency of 95% for 80 cycles. The cell under a pressure of 1.85 MPa also maintained a coulombic efficiency of 95% for 30 cycles, although it then gradually decreased. At a current density of 1.0 mA cm^−2^ (Fig. 7b), the cell without pressure maintained a coulombic efficiency of 88% for 20 cycles, but it then rapidly decreased and was no longer cycled after that. In the cell under a pressure of 0.69 MPa, the coulombic efficiency was *ca.* 93% for 40 cycles. The cells with applied pressures of 1.39 MPa and 1.85 MPa exhibited the highest coulombic efficiency of 98%. However, in contrast to the performance of the cell under 1.39 MPa, that of the cell under an applied pressure of 1.85 MPa suddenly decreased after 10 cycles. A similar tendency was observed at 2.0 mA cm^−2^ (Fig. 7c). The cell without pressure exhibited a maximum coulombic efficiency of 88%, which then quickly declined. There was little difference in the coulombic efficiency of the cells under pressures of 0.69 MPa and 1.39 MPa, which gradually decreased from 97% early in the cycle. Although cyclability of the coulombic efficiency was decreased when the current density was increased, the 0.69–1.39 MPa of pressure suppressed the degradation of plating/striping reaction at the high current density cycling. The charge/discharge curve at 0.2 mA cm^−2^ showed that application of pressure to the cell resulted in a stable voltage profile and a reduction of the charge capacity during cycling was suppressed (Fig. 7d–f). It is considered that the suppression of lithium dendrite formation and side reactions was achieved by the formation of compact and flat morphology of the lithium electrodeposited under pressure. Furthermore, polarization magnitude of the cell was estimated as the displacement of plateau voltage for lithium plating/stripping reaction from 0 V. The polarization was suppressed in the cells when a pressure below 1.39 MPa was applied. In contrast, a large polarization was observed for the cell operated under 1.85 MPa, which indicates that the applied pressure was so strong that it caused depletion of the electrolyte between the electrodes and a large interfacial resistance. These results show that an appropriate amount of applied pressure leads to improvement of the plating/stripping behavior for a lithium metal anode. Including the results of the morphological (Fig. 2) and EIS (Fig. 3) analyses, a pressure around 1.39 MPa is considered to be most effective, which approximately corresponds to the previous cycling result in anode-free lithium metal battery with carbonate electrolyte system.^39^ The electrochemical performance of the lithium plating/stripping reaction also indicated that there are different characteristics with or without uniaxial pressure in the beginning of the cycle. At the beginning of a cycle without pressure, the coulombic efficiency was gradually improved from the first cycle, whereas the highest coulombic efficiency was confirmed from the first cycle at any current density when pressure was applied.

**Fig. 8:**
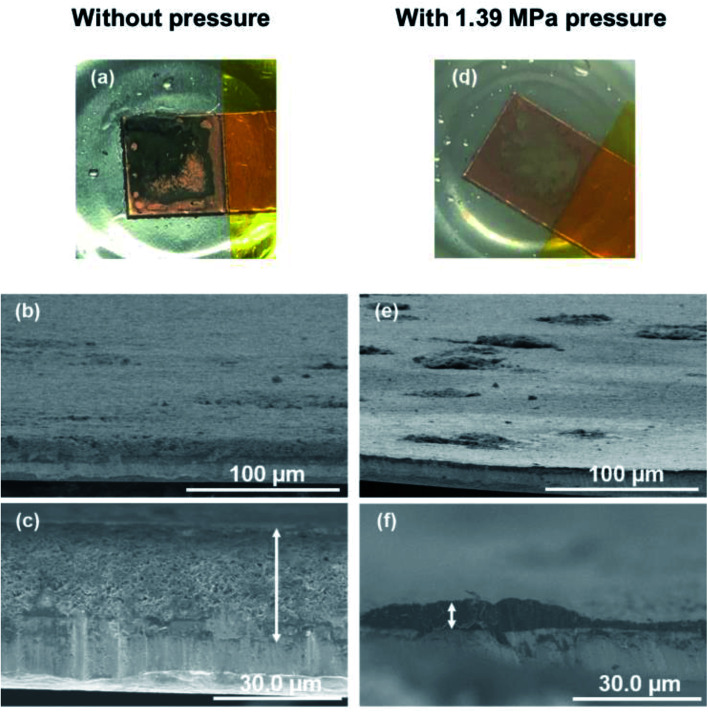
Surface optical and cross-sectional SEM images of the copper electrode after the 1^st^ plating/stripping cycle at 1.0 mA cm^−2^ for 1 h without pressure (a–c), and under a pressure of 1.39 MPa (d–f).

The morphologies of the copper cathodes operated without pressure and under a pressure of 1.39 MPa, of which the coulombic efficiencies differed significantly at the first cycle, were compared after the 1^st^ cycle at 1.0 mA cm^−2^. Fig. 8 shows the surface optical and cross-sectional SEM images of the copper electrode after the 1^st^ cycle at 1.0 mA cm^−2^ for 1 h with (1.39 MPa) and without pressure. For the copper electrode operated without pressure, the optical image revealed black deposits on the surface that could be considered to be by-products. The SEM images in Fig. 8b and c reveals the fibrous and sparse deposits consisting of by-products and undissolved dendritic lithium on the copper electrode surface. These deposits result in a large overpotential and irreversible reaction during operation without pressure. In contrast, a small amount of gray substance was thinly deposited on the copper surface when 1.39 MPa of pressure was applied. This indicates that no significant amount of undissolved lithium and by-products were present on the electrode operated under a pressure of 1.39 MPa. This morphological difference between with and without uniaxial pressure corresponds to that confirmed in the initial coulombic efficiency shown in Fig. 7. The separator is pressed against the electrode surface when the pressure is applied, which could be easily maintain the SEI layer on the electrode surface by close contact and suppress side reactions with the electrolyte by a decrease in the amount of excess electrolyte solution. Therefore, the lithium electrodeposited under uniaxial pressure was sufficiently removed, even in the first cycle, which led to a high coulombic efficiency.

### 3.4. Deflection of separator during lithium electrodeposition under pressure

**Fig. 9:**
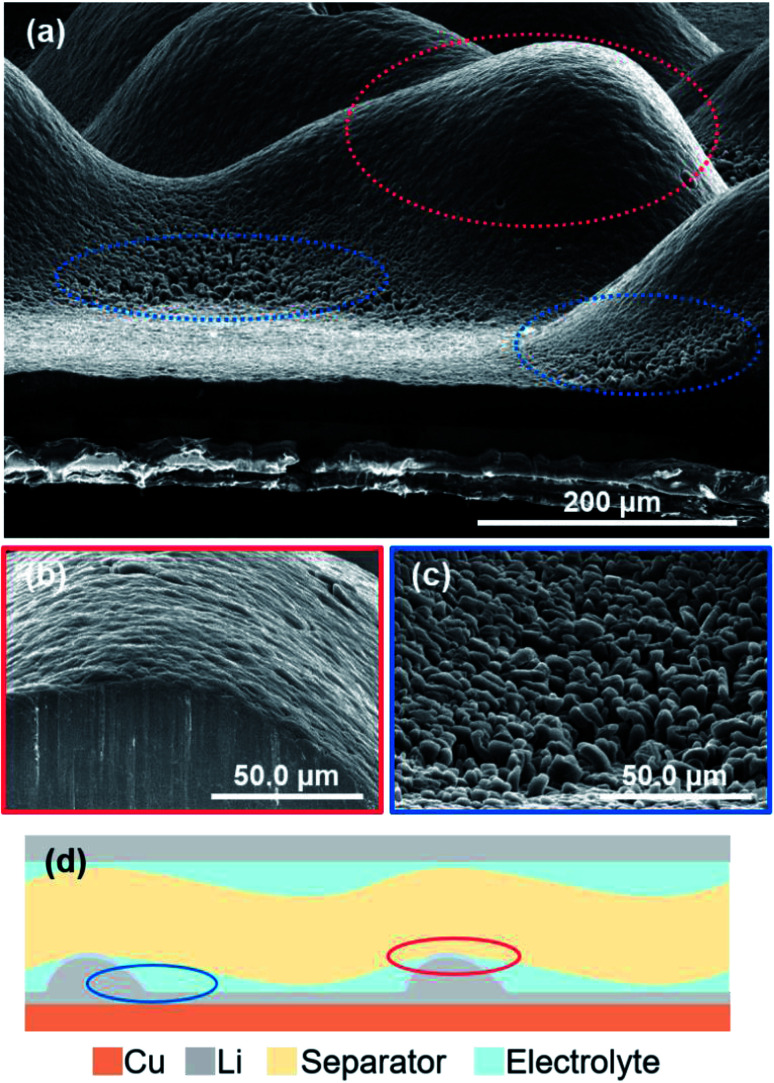
(a) Cross-sectional SEM images of the electrodeposited lithium metal after plating on the copper substrate at 15 mA h cm^−2^ under a pressure of 1.39 MPa. Expanded images at (b) the convex portion and (c) the concave portion. (d) Schematic diagram of the morphology of electrodeposited lithium in contact with a separator with poor flatness.

Fig. 9 shows cross-sectional SEM images of the electrodeposited lithium metal on the copper substrate after plating at 15 mA h cm^−2^ under a pressure of 1.39 MPa. When the amount of electrodeposition significantly increased under the pressure, the electrodeposited lithium did not become flat, but was uneven on the copper substrate. The convex surface of the plated lithium had a smooth morphology (Fig. 9b); however granular lithium growth in the direction perpendicular to the substrate was confirmed in the concave part (Fig. 9c). This indicates that the contact between electrodeposited lithium and the separator should be inhomogeneous, even under pressure, mainly because of the deflection of the separator sheet (Fig. 9d). If the separator is easily bent by the growth of lithium during deposition, it cannot restrict growth space of lithium, which leads that the applied load becomes uneven and the flatness of the deposition morphology is lost. In this case, the load from the separator is concentrated on the convex part, whereas it decreases in the surrounding area. Therefore, the surface of the electrodeposited lithium metal on the convex part becomes smooth (Fig. 9b). In contrast, a small amount of electrodeposited lithium is grown in the direction perpendicular to the copper substrate because the concave portion is less restricted by the separator. Similar morphology was also confirmed for lithium electrodeposited at current densities from 0.2 to 2.0 mA cm^−2^ under each pressure. Therefore, if the flatness of the separator is lost, even under pressure, then the limitation of the lithium growth space by the separator becomes non-uniform on the electrode. This also occurs during cycling because the morphology continues to change by the plating/stripping reaction.

**Fig. 10:**
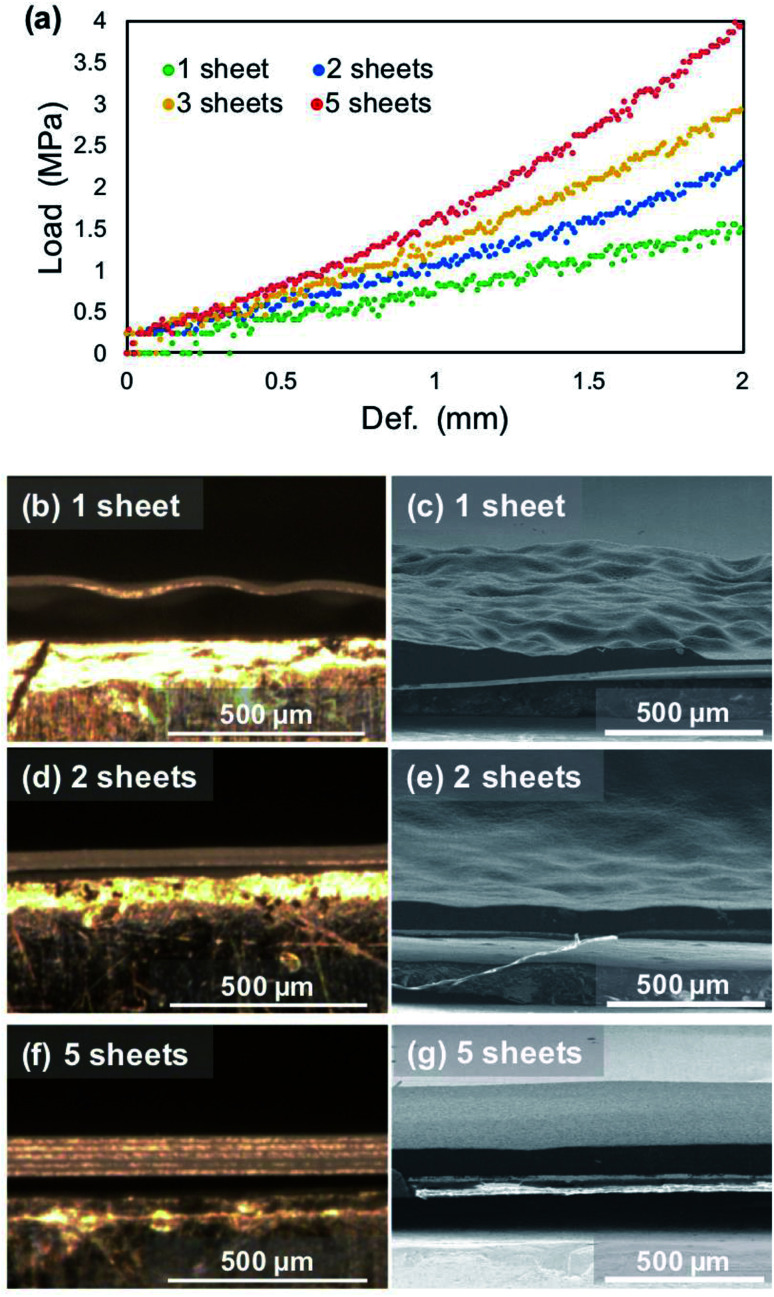
Relationship between the deflection of the separator sheet and the morphology of electrodeposited lithium. (a) Stress–strain curves as a function of the number of separators. (b, d and f) Cross-sectional optical images of separators and (c, e and g) SEM images of lithium electrodeposited on a copper substrate at 15 mA h cm^−2^ under a pressure of 1.39 MPa with various numbers of separator sheets in the cell; (b and c) one, (d and e) two and (f and g) five separator sheets.

Therefore, we examined whether a dense and flat deposition morphology and improvement of the cycle performance were realized with a layered polyethylene separator that is anticipated to have a high rigidity modulus under pressure. The initial plating proceeds under a pressure of 1.39 MPa; therefore, the separator layers between the electrodes can be regarded as a continuum, of which the thickness has changed in a pseudo manner according to the number of sheets used. Bending tests of each number of separator sheets was conducted to investigate the change in the deflection of the separators. Cross-sectional observations of the separator/electrodeposited lithium interface after 1^st^ plating was then performed. Fig. 10a shows stress–strain curves of the polyethylene separator layers measured by the three-point bending test. As the number of separators increased from 1 to 5 sheets, the reaction force against the deflection increased, which means that it is less likely to bend as the thickness of the separator sheet increases. In other words, the rigidity of the separator layer with respect to the force received from the asperities of lithium is improved. To confirm the influence of deflection, a large amount of lithium was electrodeposited on the copper electrode at 15 mA cm^−2^. Fig. 10b–g show cross-sectional optical images of the interface between the separator and electrodeposited lithium and SEM images of lithium electrodeposited on a copper substrate after the 1^st^ plating cycle (1 mA cm^−2^, 15 h). When one separator was used in the cell, it was significantly bent and its shape did not return to flat after peeling off from the electrode (Fig. 10b). Electrodeposited lithium was also densely deposited and the surface was smooth, but it was not flat on the macroscale (Fig. 10c). In contrast, when 2 or 5 separators were used (Fig. 10d and f), a dense and flat morphology of the electrodeposited lithium was achieved (Fig. 10e and g). These results suggest that the deflection of the separator is largely related to the electrodeposition of lithium. The morphology of electrodeposited lithium can be efficiently smoothed through the use of a rigid separator that is difficult to bend.

**Fig. 11:**
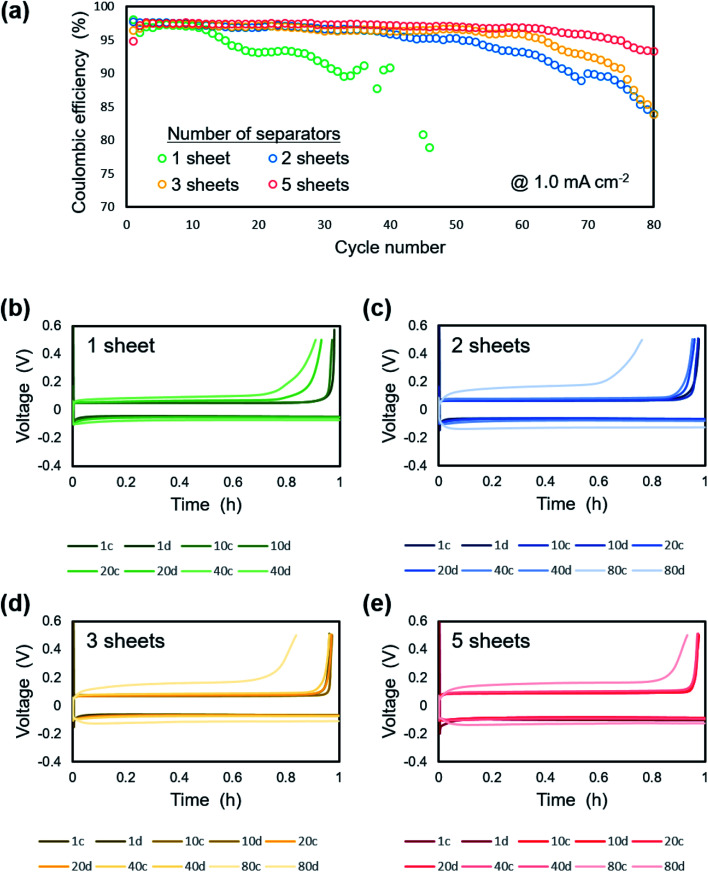
Electrochemical performance of lithium plating/stripping on copper substrate at 1.0 mA cm^−2^ under a pressure of 1.39 MPa. (a) Cycle dependence of coulombic efficiency with various numbers of separator sheets. Charge/discharge curves of Li/Cu cells with (b) 1, (c) 2, (d) 3 and, (e) 5 separator sheets.

Fig. 11 shows the electrochemical performance of lithium plating/stripping on the copper substrate at 1.0 mA cm^−2^ under a pressure of 1.39 MPa with a different number of separator sheets. There was no difference in the initial coulombic efficiency, which was *ca.* 97%. On the other hand, extended cycle life was confirmed as the number of separator sheets increased. For the cell with one separator, the coulombic efficiency gradually decreased from the 15^th^ cycle and was under 50% after 50 cycles. The cells with two or more separators maintained a coulombic efficiency of *ca.* 95% even after 50 cycles. The cells with 2 or 3 separator sheets exhibited a decrease in coulombic efficiency from around the 50^th^ and 60^th^ cycle, respectively. There was no significant change in the coulombic efficiency of the cell with 5 separator sheets during cycling and a coulombic efficiency of *ca.* 90% was maintained for 80 cycles. These results can be attributed to a more homogeneous electrodeposition with the less amount of dead lithium and electrolyte decomposition using multi-separators than that using single one. Fig. 11b–e show charge/discharge curves of Li/Cu cells with (b) 1, (c) 2, (d) 3 and, (e) 5 separator sheets. For the cell with one separator, slight polarization was confirmed after 40 cycles. As the number of separators used in the cell increased from 2 to 5 separator sheets, the overpotential increased slightly. However, an increase in overpotential with cycling was suppressed. In particular, no increase of polarization was observed for up to 40 cycles with the cell using 5 separator sheets and the polarization at 80 cycles was less than that of the cells with 2 and 3 separator sheets. Although the morphology and shape of the electrode continues to change with each charge and discharge reaction, the homogeneous load under uniaxial pressure was maintained because of the high rigidity of the layered separator, which maintained the flatness of the electrode. This leads to compact and smooth lithium electrodeposition and thus high coulombic efficiency.

## 4 Conclusion

Experimental observation indicated that application of an external pressure can effectively suppress lithium dendrite formation. The electrodeposition and electrochemical properties of lithium metal were found to change with the strength of the applied pressure. Lithium metal was deposited with a dendritic morphology when no pressure was applied, whereas a granular and dense morphology was formed under pressure. This morphological change was confirmed for pressures up to 1.39 MPa. The cycle performance and coulombic efficiency was improved when the applied pressure was below 1.39 MPa. In addition, the flatness of electrodeposited lithium was confirmed to be strongly related to the rigidity of the separator layer. The morphology of electrodeposited lithium became flatter with a large amount of electrodeposition under pressure when the number of polyethylene separator sheets was increased to five because of the increase in the stiffness of the layered separator. High coulombic efficiency cycling by pressurization was maintained twice as long as that for one separator sheet. Therefore, the use of a high rigidity separator and application of an appropriate amount of pressure are effective approaches to control lithium growth and improve the performance of lithium metal batteries.

## Conflicts of interest

There are no conflicts to declare.

## Supplementary Material
